# Gene expression microarray data from human microvascular endothelial cells supplemented with a low concentration of niacin

**DOI:** 10.1016/j.dib.2016.01.039

**Published:** 2016-02-03

**Authors:** Jennifer M. Hughes-Large, Nica M. Borradaile

**Affiliations:** Department of Physiology and Pharmacology, Schulich School of Medicine and Dentistry, Western University, London, Ontario, Canada

**Keywords:** Vascular biology, Endothelial cells, Niacin, Lipids

## Abstract

The systemic lipid modifying drug, niacin, can directly improve human microvascular endothelial cell angiogenic function under lipotoxic conditions, possibly through activation of niacin receptors “Niacin receptor activation improves human microvascular endothelial cell angiogenic function during lipotoxicity” (Hughes-Large et al. 2014). Here we provide accompanying data collected using Affymetrix GeneChip microarrays to identify changes in gene expression in human microvascular endothelial cells treated with 10 μM niacin. Statistical analyses of robust multi-array average (RMA) values revealed that only 16 genes exhibited greater than 1.3-fold differential expression. Of these 16, only 5 were identified protein coding genes, while 3 of the remaining 11 genes appeared to be small nuclear/nucleolar RNAs. Altered expression of *EFCAB4B*, *NAP1L2*, and *OR13C8* was confirmed by real time quantitative PCR.

**Specifications table**

**Table:** 

Subject area	Vascular biology
More specific subject area	Endothelial cell biology
Type of data	Tables
How data was acquired	Affymetrix GeneChip RNA Microarray, RMA and statistical analyses, real time quantitative PCR
Data format	Filtered, analyzed
Experimental factors	Human microvascular endothelial cells were incubated with growth media containing either vehicle control (water) or niacin for 24 h.
Experimental features	RNA isolation, global gene expression analyses, and real time quantitative PCR.
Data source location	London, Ontario, Canada
Data accessibility	Data is within this article.

**Value of the data**

• A global gene expression analysis of human endothelial cells treated with niacin.
• These data may be useful for comparison with microarray data from other cell or tissue types treated with niacin.
• Genes identified as differentially expressed in this data set could be included in further studies of the direct effects of niacin on the vasculature.

## 1 Data

Affymetrix GeneChip microarray analyses of mRNA isolated from human microvascular endothelial cells (HMVEC) following 24 h treatment with 10 μM niacin revealed significantly (*P*<0.05) altered expression of only five protein coding genes at a fold change of greater than 1.3 (Table 1). Changes in the expression of *EFCAB4B, NAP1L2*, and *OR13C8* in response to niacin treatment were confirmed by real time quantitative PCR. We also observed altered expression (>1.3 fold, *P*<0.05) of 3 non-coding sequences in HMVEC treated with niacin (Table 2), which appear to be small nuclear or nucleolar RNAs (snRNA and snoRNA). Our experiment was conducted using arrays consisting of predominantly coding transcripts (GeneChip Human Gene 1.0 ST, Affymetrix), and was not optimized to rigorously detect changes in the expression of small non-coding sequences.

## 2 Experimental design, materials and methods

### 2.1 Endothelial cell culture and treatments

Primary HMVEC (Lonza) were maintained in Medium 199 (Life Technologies) supplemented with EGM-2MV SingleQuots (Lonza), and subcultured as recommended by the supplier. For experiments, cells from three independent subcultures from a single donor were used. Cell monolayers at 80% confluence were incubated for 24 h with experimental media supplemented with either cell culture grade water as vehicle control (Life Technologies) or 10 μM niacin (Fluka BioChemika) solubilized in cell culture grade water. A total of six samples (three vehicle control, three niacin treated) were generated for subsequent gene expression analyses.

### 2.2 RNA Isolation, quality assessment, probe preparation and GeneChip hybridization

Total RNA was prepared as previously described [1]. Cell monolayers were harvested using trypsin and lysed with QIAshredder columns (Qiagen). Total RNA was isolated using an RNeasy Mini Kit (Qiagen), and eluted with nuclease-free water. RNA was stored at −80 °C for 1 week prior to microarray analyses.

All subsequent sample handling, labeling, and GeneChip (Human Gene 1.0 ST arrays) processing was performed at the London Regional Genomics Center (Robarts Research Institute, London, Ontario, Canada; http://www.lrgc.ca). RNA quality was assessed using an Agilent 2100 Bioanalyzer (Agilent Technologies Inc., Palo Alto, CA) and the RNA 6000 Nano kit (Caliper Life Sciences, Mountain View, CA). Single stranded complimentary DNA was prepared from 200 ng of total RNA as per the Ambion WT Expression Kit for Affymetrix GeneChip Whole Transcript WT Expression Arrays (Applied Biosystems and Affymetrix). Total RNA was first converted to cDNA, followed by in vitro transcription to make cRNA. Single stranded cDNA was synthesized, end labeled and hybridized, for 16 h at 45 °C, to Human Gene 1.0 ST arrays (Affymetrix). All liquid handling steps were performed by a GeneChip Fluidics Station 450 and GeneChips were scanned with the GeneChip Scanner 3000 7G (Affymetrix) using Command Console v3.2.4.

### 2.3 Statistical analyses of changes in global gene expression

All microarray data complies with MIAME guidelines. Probe level data was generated using Affymetrix Command Console v3.2.4. Probes were summarized to gene level data in Partek Genomics Suite v6.6 (Partek) using the robust multi-array average (RMA) algorithm [2]. Partek was used to determine gene level ANOVA *P*-values and fold changes. A filtered gene list was generated for expression changes of least 1.3 fold and having a *P*-value of less than 0.05 (Supplementary material).

### 2.4 Real time quantitative PCR

Selected changes in gene expression were independently assessed by real time quantitative PCR (qRT-PCR). cDNA was synthesized from three independent RNA samples each from vehicle control and niacin-treated cells (prepared as described above) using a High Capacity RNA-to-cDNA kit (Applied Biosystems). TaqMan gene expression assays for *GAPDH* (internal control), *EFCAB4B* (Hs01592234_m1), *NAP1L2* (Hs01114608_s1), and *OR13C8* (Hs01104244_s1) were from Applied Biosystems. All reactions were performed according to protocols provided by the supplier using a 7900 HT Fast Real-Time PCR System (Applied Biosystems). Cycle threshold (Ct) values were used to calculate the relative quantification (RQ) for each sample and fold changes for each gene.

## Figures and Tables

**Table 1:** Altered coding gene expression in HMVEC treated with niacin.

Direction of regulation	Gene symbol	Function	*P*-value	Fold change (microarray)	Fold change (qRT-PCR)
Down	*EFCAB4B*	Ca^2+^ regulation during inflammation	0.00165	−1.31930	−1.41
	*NAP1L2*	Chromatin modification	0.00481	−1.34332	−1.27
Up	*OR13C8*	Unknown	0.01870	1.35805	1.38
	*SIGLEC5*	Cell–cell interactions	0.02342	1.32142	ND
	*CYP4Z1*	Tumor angiogenesis	0.02577	1.35765	ND

HMVEC were incubated for 24 h with growth medium containing either vehicle (water), or 10 μM niacin. Total RNA was extracted and expression microarray analyses were performed. Transcripts with fold changes >1.3 (niacin vs. vehicle control) for 3 independent experiments are shown. Fold changes were confirmed by qRT-PCR where indicated, *n*=3; ND, not determined.

**Table 2:** Altered small non-coding gene expression in HMVEC treated with niacin.

Direction of regulation	mRNA assignment	*P*-value	Fold change (microarray)
Down	snRNA chromosome:GRCh37:3:40540382:40540494	0.04607	−1.30616
Up	*SNORD13P1*	0.00821	1.43638
	snoRNA pseudogene chromosome:GRCh37:X:13	0.02069	1.41598

HMVEC were incubated for 24 h with growth medium containing either vehicle (water), or 10 μM niacin. Total RNA was extracted and expression microarray analyses were performed. Transcripts with fold changes >1.3 (niacin vs. vehicle control) for 3 independent experiments are shown.
