# Supplementary material for: Gene expression microarray data from human microvascular endothelial cells supplemented with a low concentration of niacin
Source: Data Brief. 2016 Feb 3;6:899–902. doi: 10.1016/j.dib.2016.01.039 (PMC4752733; doi:10.1016/j.dib.2016.01.039)
Supplement: Supplementary file 1 — Supplementary material [file mmc1.pdf]

Column #	Transcript ID	Gene Assignment	Gene Symbol	RefSeq	mRNA Assignment	*P* Value (Niacin vs. H2O)	Ratio (Niacin vs. H2O)	Fold-Change (Niacin vs. H2O)
1364	7893862	---		---	--- // --- // neg_control // --- // --- // --- // --- // --- // ---	0.0297899	0.38962	-2.56661
2825	7895335	---		---	--- // --- // neg_control // --- // --- // --- // --- // --- // ---	0.0395318	0.470365	-2.12601
720	7893216	---		---	--- // --- // neg_control // --- // --- // --- // --- // --- // ---	0.0353339	0.530094	-1.88646
4099	7896619	---		---	--- // --- // neg_control // --- // --- // --- // --- // --- // ---	0.00210939	0.547737	-1.82569
2840	7895350	---		---	--- // --- // neg_control // --- // --- // --- // --- // --- // ---	0.0171862	0.552917	-1.80859
1651	7894151	---		---	--- // --- // neg_control // --- // --- // --- // --- // --- // ---	0.0209839	0.562464	-1.77789
468	7892962	---		---	--- // --- // neg_control // --- // --- // --- // --- // --- // ---	0.00908661	0.570747	-1.75209
21863	8073680	---		---	ENST00000466665 // ENSEMBL // ncrna_pseudogene:tRNA_pseudogene chromosome:GRCh37:22:445	0.0404452	0.575132	-1.73873
3886	7896402	---		---	--- // --- // neg_control // --- // --- // --- // --- // --- // ---	0.00758485	0.600554	-1.66513
1969	7894473	---		---	--- // --- // neg_control // --- // --- // --- // --- // --- // ---	0.00510742	0.613536	-1.6299
694	7893190	---		---	--- // --- // neg_control // --- // --- // --- // --- // --- // ---	0.0145421	0.615361	-1.62506
3924	7896441	---		---	--- // --- // neg_control // --- // --- // --- // --- // --- // ---	0.0113279	0.617212	-1.62019
2278	7894786	---		---	--- // --- // neg_control // --- // --- // --- // --- // --- // ---	0.043544	0.623198	-1.60463
4087	7896607	---		---	--- // --- // neg_control // --- // --- // --- // --- // --- // ---	0.0493568	0.632386	-1.58131
3245	7895756	---		---	--- // --- // neg_control // --- // --- // --- // --- // --- // ---	0.0492107	0.63392	-1.57749
1088	7893584	---		---	--- // --- // neg_control // --- // --- // --- // --- // --- // ---	0.0208605	0.635723	-1.57301
1582	7894081	---		---	--- // --- // neg_control // --- // --- // --- // --- // --- // ---	0.0406529	0.643198	-1.55473
391	7892885	---		---	--- // --- // neg_control // --- // --- // --- // --- // --- // ---	0.0141893	0.653139	-1.53107
1046	7893542	---		---	--- // --- // neg_control // --- // --- // --- // --- // --- // ---	0.0136475	0.658435	-1.51875
19068	8045205	NR_027313 // LOC150527 // hypothetical LOC150527 // 2q21.1 // 150527 /// NR_033930 // L	LOC150527	NR_027313	NR_027313 // RefSeq // Homo sapiens hypothetical LOC150527 (LOC150527), transcript vari	0.0131346	0.661192	-1.51242
20051	8055236	NR_027313 // LOC150527 // hypothetical LOC150527 // 2q21.1 // 150527 /// NR_033930 // L	LOC150527	NR_027313	NR_027313 // RefSeq // Homo sapiens hypothetical LOC150527 (LOC150527), transcript vari	0.0131346	0.661192	-1.51242
3843	7896359	---		---	--- // --- // neg_control // --- // --- // --- // --- // --- // ---	0.0416256	0.661908	-1.51078
4139	7896659	---		---	--- // --- // pos_control // --- // --- // --- // --- // --- // ---	0.0127423	0.664078	-1.50585
3277	7895789	---		---	--- // --- // neg_control // --- // --- // --- // --- // --- // ---	0.00948824	0.666077	-1.50133
3576	7896092	---		---	--- // --- // neg_control // --- // --- // --- // --- // --- // ---	0.00874762	0.667459	-1.49822
2618	7895128	---		---	--- // --- // neg_control // --- // --- // --- // --- // --- // ---	0.0351104	0.670257	-1.49196
1013	7893509	---		---	--- // --- // neg_control // --- // --- // --- // --- // --- // ---	0.0241457	0.672317	-1.48739
5261	7907010	---		---	ENST00000434461 // ENSEMBL // cdna:pseudogene chromosome:GRCh37:1:166535415:166549942:1	0.0418884	0.672834	-1.48625
957	7893453	---		---	--- // --- // pos_control // --- // --- // --- // --- // --- // ---	0.045419	0.673211	-1.48542
27013	8124057	---		---	ENST00000439284 // ENSEMBL // cdna:known chromosome:GRCh37:6:17531125:17531523:-1 gene:	0.0464883	0.676725	-1.47771
1689	7894190	---		---	--- // --- // neg_control // --- // --- // --- // --- // --- // ---	0.0411741	0.676775	-1.4776
3469	7895983	---		---	--- // --- // neg_control // --- // --- // --- // --- // --- // ---	0.036507	0.677125	-1.47683
1993	7894497	---		---	--- // --- // neg_control // --- // --- // --- // --- // --- // ---	0.0357329	0.683103	-1.46391
2089	7894594	---		---	--- // --- // pos_control // --- // --- // --- // --- // --- // ---	0.0124196	0.692306	-1.44445
2664	7895174	---		---	--- // --- // neg_control // --- // --- // --- // --- // --- // ---	0.0137303	0.69764	-1.4334
1191	7893688	---		---	--- // --- // neg_control // --- // --- // --- // --- // --- // ---	0.0109596	0.697807	-1.43306
1696	7894197	---		---	--- // --- // neg_control // --- // --- // --- // --- // --- // ---	0.0478621	0.698027	-1.43261
1841	7894343	---		---	--- // --- // neg_control // --- // --- // --- // --- // --- // ---	0.00619406	0.702572	-1.42334
32711	8176815	---		---	ENST00000400578 // ENSEMBL // cdna:known chromosome:GRCh37:Y:24795438:24800925:1 gene:E	0.00724267	0.704364	-1.41972
32725	8176941	---		---	ENST00000400578 // ENSEMBL // cdna:known chromosome:GRCh37:Y:24795438:24800925:1 gene:E	0.00724267	0.704364	-1.41972
32795	8177393	---		---	ENST00000400578 // ENSEMBL // cdna:known chromosome:GRCh37:Y:24795438:24800925:1 gene:E	0.00724267	0.704364	-1.41972
125	7892619	---		---	--- // --- // neg_control // --- // --- // --- // --- // --- // ---	0.0176345	0.713001	-1.40252
428	7892922	---		---	--- // --- // neg_control // --- // --- // --- // --- // --- // ---	0.00994509	0.723053	-1.38302
3364	7895876	---		---	--- // --- // neg_control // --- // --- // --- // --- // --- // ---	0.0403483	0.731777	-1.36654
3580	7896096	---		---	--- // --- // neg_control // --- // --- // --- // --- // --- // ---	0.0263675	0.732783	-1.36466
564	7893060	---		---	--- // --- // neg_control // --- // --- // --- // --- // --- // ---	0.00281154	0.737791	-1.3554
1499	7893998	---		---	--- // --- // neg_control // --- // --- // --- // --- // --- // ---	0.033886	0.739159	-1.35289
1057	7893553	---		---	--- // --- // neg_control // --- // --- // --- // --- // --- // ---	0.0290997	0.739814	-1.35169
645	7893141	---		---	--- // --- // neg_control // --- // --- // --- // --- // --- // ---	0.0426385	0.742416	-1.34695
28988	8143038	---		---	ENST00000484390 // ENSEMBL // ncrna_pseudogene:tRNA_pseudogene chromosome:GRCh37:7:1331	0.0406545	0.743239	-1.34546
32314	8173600	NM_021963 // NAP1L2 // nucleosome assembly protein 1-like 2 // Xq13 // 4674 /// ENST000	NAP1L2	NM_021963	NM_021963 // RefSeq // Homo sapiens nucleosome assembly protein 1-like 2 (NAP1L2), mRNA	0.00480989	0.744422	-1.34332
1476	7893975	---		---	--- // --- // neg_control // --- // --- // --- // --- // --- // ---	0.0246985	0.747385	-1.338
10285	7956747	---		---	AL833331 // GenBank // Homo sapiens mRNA; cDNA DKFZp686H1233 (from clone DKFZp686H1233)	0.024885	0.747656	-1.33751
4093	7896613	---		---	--- // --- // neg_control // --- // --- // --- // --- // --- // ---	0.0332773	0.753806	-1.3266
778	7893274	---		---	--- // --- // neg_control // --- // --- // --- // --- // --- // ---	0.0410361	0.755286	-1.324
1094	7893590	---		---	--- // --- // neg_control // --- // --- // --- // --- // --- // ---	0.0339398	0.755718	-1.32325
10606	7960381	CR627161 // EFCAB4B // EF-hand calcium binding domain 4B // 12p13.32 // 84766	EFCAB4B	CR627161	CR627161 // GenBank HTC // Homo sapiens mRNA; cDNA DKFZp686G13246 (from clone DKFZp686G	0.00165185	0.757975	-1.3193
22668	8081943	---		---	ENST00000383994 // ENSEMBL // ncrna:misc_RNA chromosome:GRCh37:3:119929167:119929268:1	0.0108556	0.759297	-1.31701
23104	8086350	---		---	ENST00000363036 // ENSEMBL // ncrna:snRNA chromosome:GRCh37:3:40540382:40540494:-1 gene	0.0460697	0.765603	-1.30616
17292	8028117	---		---	---	0.00103201	0.767407	-1.30309
2413	7894922	---		---	--- // --- // neg_control // --- // --- // --- // --- // --- // ---	0.0370597	1.30109	1.30109
18371	8038877	NM_003830 // SIGLEC5 // sialic acid binding Ig-like lectin 5 // 19q13.3 // 8778 /// ENS	SIGLEC5	NM_003830	NM_003830 // RefSeq // Homo sapiens sialic acid binding Ig-like lectin 5 (SIGLEC5), mRN	0.0234212	1.32142	1.32142
1842	7894344	---		---	--- // --- // neg_control // --- // --- // --- // --- // --- // ---	0.0179099	1.33477	1.33477
2200	7894707	---		---	--- // --- // neg_control // --- // --- // --- // --- // --- // ---	0.0336584	1.3438	1.3438
2756	7895266	---		---	--- // --- // neg_control // --- // --- // --- // --- // --- // ---	0.0472679	1.35525	1.35525
4656	7901287	NM_178134 // CYP4Z1 // cytochrome P450, family 4, subfamily Z, polypeptide 1 // 1p33 //	CYP4Z1	NM_178134	NM_178134 // RefSeq // Homo sapiens cytochrome P450, family 4, subfamily Z, polypeptide	0.0257688	1.35765	1.35765
30541	8157014	NM_001004483 // OR13C8 // olfactory receptor, family 13, subfamily C, member 8 // 9q31.	OR13C8	NM_001004483	NM_001004483 // RefSeq // Homo sapiens olfactory receptor, family 13, subfamily C, memb	0.0186962	1.35805	1.35805
3137	7895648	---		---	--- // --- // neg_control // --- // --- // --- // --- // --- // ---	0.0411041	1.38655	1.38655
361	7892855	---		---	--- // --- // neg_control // --- // --- // --- // --- // --- // ---	0.0146604	1.38683	1.38683
22634	8081546	---		---	ENST00000364556 // ENSEMBL // ncrna:misc_RNA chromosome:GRCh37:3:110445868:110445968:1	0.011272	1.41528	1.41528
32522	8175315	---		---	ENST00000461224 // ENSEMBL // ncrna_pseudogene:snoRNA_pseudogene chromosome:GRCh37:X:13	0.0206889	1.41598	1.41598
3452	7895966	---		---	--- // --- // neg_control // --- // --- // --- // --- // --- // ---	0.0385211	1.41641	1.41641
3122	7895633	---		---	--- // --- // neg_control // --- // --- // --- // --- // --- // ---	0.0484916	1.42434	1.42434
3042	7895553	---		---	--- // --- // neg_control // --- // --- // --- // --- // --- // ---	0.031612	1.42876	1.42876
696	7893192	---		---	--- // --- // neg_control // --- // --- // --- // --- // --- // ---	0.00501932	1.43598	1.43598
22162	8076415	X58061 // SNORD13P1 // small nucleolar RNA, C/D box 13 pseudogene 1 // 22q13.2 // 6076	SNORD13P1	X58061	X58061 // GenBank // H.sapiens U13 snRNA pseudogene U13.12A. // chr22 // 100 // 100 //	0.00821094	1.43638	1.43638
960	7893456	---		---	--- // --- // neg_control // --- // --- // --- // --- // --- // ---	0.0347554	1.43938	1.43938
3326	7895838	---		---	--- // --- // neg_control // --- // --- // --- // --- // --- // ---	0.00612493	1.45388	1.45388
2838	7895348	---		---	--- // --- // neg_control // --- // --- // --- // --- // --- // ---	0.0251312	1.45796	1.45796
3023	7895534	---		---	--- // --- // neg_control // --- // --- // --- // --- // --- // ---	0.0067228	1.47351	1.47351
2793	7895303	---		---	--- // --- // neg_control // --- // --- // --- // --- // --- // ---	0.0194446	1.47828	1.47828
1104	7893600	---		---	--- // --- // neg_control // --- // --- // --- // --- // --- // ---	0.0252411	1.49661	1.49661
2033	7894537	---		---	--- // --- // neg_control // --- // --- // --- // --- // --- // ---	0.0121128	1.50372	1.50372
1942	7894446	---		---	--- // --- // neg_control // --- // --- // --- // --- // --- // ---	0.041838	1.50781	1.50781
1244	7893741	---		---	--- // --- // neg_control // --- // --- // --- // --- // --- // ---	0.01815	1.5194	1.5194
1567	7894066	---		---	--- // --- // neg_control // --- // --- // --- // --- // --- // ---	0.00337906	1.56029	1.56029
1522	7894021	---		---	--- // --- // neg_control // --- // --- // --- // --- // --- // ---	0.0360577	1.59354	1.59354
2772	7895282	---		---	--- // --- // neg_control // --- // --- // --- // --- // --- // ---	0.0106006	1.59683	1.59683
2673	7895183	---		---	--- // --- // neg_control // --- // --- // --- // --- // --- // ---	0.0425688	1.63789	1.63789
3936	7896453	---		---	--- // --- // neg_control // --- // --- // --- // --- // --- // ---	0.0261545	1.68323	1.68323
728	7893224	---		---	--- // --- // neg_control // --- // --- // --- // --- // --- // ---	0.0252514	1.70794	1.70794
31512	8166607	---		---	ENST00000496936 // ENSEMBL // ncrna_pseudogene:rRNA_pseudogene chromosome:GRCh37:X:2898	0.00355907	1.71948	1.71948
3065	7895576	---		---	--- // --- // neg_control // --- // --- // --- // --- // --- // ---	0.0302395	1.81988	1.81988
3096	7895607	---		---	--- // --- // neg_control // --- // --- // --- // --- // --- // ---	0.0443572	1.90029	1.90029
416	7892910	---		---	--- // --- // neg_control // --- // --- // --- // --- // --- // ---	0.0389976	2.07536	2.07536
16599	8021368	---		---	ENST00000482366 // ENSEMBL // ncrna_pseudogene:tRNA_pseudogene chromosome:GRCh37:18:553	0.0304355	2.14336	2.14336
2360	7894869	---		---	--- // --- // neg_control // --- // --- // --- // --- // --- // ---	0.0192379	2.16318	2.16318
2270	7894778	---		---	--- // --- // neg_control // --- // --- // --- // --- // --- // ---	0.0124079	2.19065	2.19065
2903	7895413	---		---	--- // --- // neg_control // --- // --- // --- // --- // --- // ---	0.0428207	2.23534	2.23534
115	7892609	---		---	--- // --- // pos_control // --- // --- // --- // --- // --- // ---	0.00272071	3.78636	3.78636
